# A prospective observational cohort study in primary care practices to identify factors associated with treatment failure in *Staphylococcus aureus* skin and soft tissue infections

**DOI:** 10.1186/s12941-016-0175-8

**Published:** 2016-11-22

**Authors:** Grace C. Lee, Ronald G. Hall, Natalie K. Boyd, Steven D. Dallas, Liem C. Du, Lucina B. Treviño, Sylvia B. Treviño, Chad Retzloff, Kenneth A. Lawson, James Wilson, Randall J. Olsen, Yufeng Wang, Christopher R. Frei

**Affiliations:** 1 College of Pharmacy, University of Texas at Austin, Austin, TX USA; 2 Pharmacotherapy Education and Research Center, School of Medicine, The University of Texas Health Science Center, 7703 Floyd Curl Dr, MC 6220, San Antonio, TX 78229-3900 USA; 3 School of Pharmacy, Texas Tech University Health Sciences Center, Dallas, TX USA; 4 Dose Optimization and Outcomes Research (DOOR) Program, Dallas, TX USA; 5 Department of Clinical Laboratory Sciences, School of Health Professions, University of Texas Health Science Center, San Antonio, TX USA; 6 South Texas Ambulatory Research Network, The University of Texas Health Science Center, San Antonio, TX USA; 7 Department of Pathology and Genomic Medicine, Houston Methodist Hospital and Research Institute, Houston, TX USA; 8 Department of Biology, The University of Texas San Antonio, San Antonio, TX USA

**Keywords:** *Staphylococcus aureus*, Skin and soft tissue infections, Methicillin-resistant *Staphylococcus aureus* (MRSA), Epidemiology, Primary care

## Abstract

**Background:**

The incidence of outpatient visits for skin and soft tissue infections (SSTIs) has substantially increased over the last decade. The emergence of community-associated methicillin-resistant *Staphylococcus aureus* (CA-MRSA) has made the management of *S. aureus* SSTIs complex and challenging. The objective of this study was to identify risk factors contributing to treatment failures associated with community-associated *S. aureus* skin and soft tissue infections SSTIs.

**Methods:**

This was a prospective, observational study among 14 primary care clinics within the South Texas Ambulatory Research Network. The primary outcome was treatment failure within 90 days of the initial visit. Univariate associations between the explanatory variables and treatment failure were examined. A generalized linear mixed-effect model was developed to identify independent risk factors associated with treatment failure.

**Results:**

Overall, 21% (22/106) patients with *S. aureus* SSTIs experienced treatment failure. The occurrence of treatment failure was similar among patients with methicillin-resistant *S. aureus* and those with methicillin-susceptible *S. aureus* SSTIs (19 vs. 24%; *p* = 0.70). Independent predictors of treatment failure among cases with *S. aureus* SSTIs was a duration of infection of ≥7 days prior to initial visit [aOR, 6.02 (95% CI 1.74–19.61)] and a lesion diameter size ≥5 cm [5.25 (1.58–17.20)].

**Conclusions:**

Predictors for treatment failure included a duration of infection for ≥7 days prior to the initial visit and a wound diameter of ≥5 cm. A heightened awareness of these risk factors could help direct targeted interventions in high-risk populations.

## Background

The incidence of outpatient and emergency department visits for skin and soft tissue infections (SSTIs) has substantially increased with the emergence of community-associated methicillin-resistant *Staphylococcus aureus* (CA-MRSA) [1]. In the U.S., approximately 80–90% of SSTIs are due to *S. aureus* [2, 3]. Moreover, treatment failure is common after an initial *S. aureus* SSTI episode; recurrence rates have exceeded 50% in some populations [4]. Treatment failure may be multifactorial and can be associated with host factors, disease management, and pathogen features. Two studies set in urgent care and primary care clinics found 35% of patients with CA-MRSA SSTIs experienced treatment failure and 78% reported SSTI recurrence [5, 6]. SSTIs due to CA-MRSA have been implicated to have more serious outcomes compared to community-associated methicillin susceptible *S. aureus* (CA-MSSA) SSTIs; however, there are limited studies evaluating the differences in treatment outcomes in the primary care setting. Furthermore, while there has been a growing body supporting the assessment of early response in treatment failure among hospitalized patients with SSTIs, very little information is available for outpatients [7, 8]. Tools to better identify those who are at higher risk of experiencing treatment failure are needed to better inform treatment decisions in the outpatient setting.

We have recently described the prevalence, treatment characteristics, and costs associated with the management of CA-MRSA SSTIs in South Texas in the primary care setting [9, 10]. The primary objective of this study was to identify risk factors contributing to *S. aureus* SSTI treatment failures.

## Methods

### Study setting and population

We performed this investigation among a well-described cohort of patients with SSTIs in the primary care setting. Details of this cohort have been described previously [9–11]. Briefly, this study was conducted in collaboration with fourteen clinics within the South Texas Ambulatory Research Network (STARNet), a practice-based research network (PBRN) composed of 108 urban, suburban, and rural primary care clinics distributed throughout the South Texas region, from 2007 to 2014. Patients were eligible for study enrollment if they provided informed consent, were 18 years of age or older, and presented to one of the participating clinics with an SSTI. Healthcare providers collected a wound sample and patient information (e.g., demographics, infection characteristics, clinical information).

### Study design

We conducted a prospective observational cohort study to determine predictors of treatment failure. Currently, there is no consensus definition of treatment failure. We have based our definition based on prior studies using a proxy of therapeutic endpoints for SSTIs in the outpatient setting [9–14]. Treatment failure was defined as any of the following events within 90 days of their initial visit: (1) need for a new course or change in antibiotic therapy for SSTI, (2) additional incision and drainage, (3) SSTI at a new site, (4) SSTI at the same site, (5) emergency department visit, or (6) hospital admission. First, we compared the rate of treatment failure for patients with MRSA infections to those with MSSA infections. Next, we identified independent risk factors associated with treatment failure by comparing key characteristics in those patients who did and did not experience treatment failure.

### Microbiologic analyses

Samples were plated onto blood agar plates (TSA with 5% sheep blood; Fisher Scientific, Lenexa, KS) and incubated at 35–37 °C for 24 h, then sub-cultured to MRSA selective agar (MRSASelect chromogenic agar plates; Bio-Rad Laboratories, Hercules, CA). Latex agglutination tests (StaphAurex^®^; Thermo Fisher Scientific, Lenexa, KS), and phenotypic screening tests (cefoxitin) were used for the identification and isolation of MRSA using Vitek 2 AST-GP75 cards (bioMerieux, Durham, NC). Antimicrobial minimum inhibitory concentrations (MICs) were interpreted according to the Clinical and Laboratory Standards Institute document M100-S22 (2012). Multidrug resistance (MDR) was defined as resistant to >2 antimicrobial classes. For molecular analysis, multilocus sequence types (MLST) were assigned for 98 isolates using whole genome sequence data according to designated MLST (http://www.mlst.net) as described previously [11].

### Data collection and variables

Clinical information included patient gender, race (Black, White, Other), ethnicity (Hispanic, Non-Hispanic), past medical history (e.g., diabetes, peripheral vascular disease, chronic non-infectious skin disorder, HIV/AIDS, cancer, actively receiving chemotherapy, immunosuppression), health care-related work history, skin infection history, height, infection characteristics (e.g., location, duration, size, deepest tunnel depth, erythema, smell, ulceration, drainage, abscess, satellites), incision and drainage procedures, and antibiotics prescribed. A BMI ≥30 kg/m^2^ was used to indicate obesity status. A 110 kg weight cutoff was used to indicate ‘high body weight’. This is consistent with previous literature associating high body weight with antimicrobial dosing outcomes [15–17]. In addition, this cut-off was internally derived using a Classification and Regression Tree (CART) analysis which found a significant node at 110 kg that partitioned the data associated with treatment failure.

### Statistical analyses

First, a bivariable analysis was conducted comparing variables between the ‘treatment failure’ and ‘no treatment failure’ groups. The Breslow-Day test was used to identify possible effect modification; any *p* ≤ 0.05 was considered an effect modifier. A generalized linear mixed-effect model was developed to identify independent risk factors; clinic site was set as the random effect. Covariates included MRSA phenotype, largest diameter size of the wound ≥5 cm, and duration of skin infection prior to visit of ≥7 days. Adjusted odds ratios (ORs) and 95% confidence intervals (CIs) were reported. A *p* ≤ 0.05 was used to determine statistical significance. SPSS 22.0^®^ (IBM Corp, Armonk, NY) was used for all statistical comparisons.

## Results

Among cases with positive *S. aureus* SSTIs, 106 cases (61%) had 90-days follow-up information. Overall, 22 (21%) cases experienced treatment failure. Treatment failure occurred in 19% (13) of cases with initial MRSA SSTIs and 24% (9) with MSSA SSTIs (*p* = 0.70). In bivariable analysis, factors associated with treatment failure included Black race, weight ≥110 kg, MDR, duration of skin infection prior to visit ≥7 days, lesion diameter ≥5 cm, lesion size ≥25 cm^2^, and abscess formation (Table 1). Multivariable analysis showed no significant difference in the likelihood of treatment failure between MRSA and MSSA (aOR, 0.42 (0.12–1.42); *p* = 0.16). Independent predictors of treatment failure among cases with *S. aureus* SSTIs were duration of skin infection prior to visit ≥7 days [aOR, 6.02 (95% CI 1.74–19.61)], and a lesion diameter size ≥5 cm [aOR, 5.25 (95% CI 1.58–17.20)].

**Table 1:** Risk factors associated with treatment failure among patients with community-associated *S. aureus* skin and soft tissue infections

Characteristic	Overall, n = 106	No failure, n = 84	Treatment failure, n = 22	OR (95% CI)	*p*	aOR (95% CI)	*p*
Mean age, years (±SD)	41 (±14)	40 (±13)	45 (±13)		0.15		
Gender							
Male	53 (50%)	44 (52%)	9 (41%)	0.63 (0.24–1.63)	0.34		
Race/ethnicity							
Black	8 (8%)	4 (5%)	4 (18%)	4.44 (1.02–19.47)	0.03		
Hispanic	78 (74%)	64 (76%)	14 (64%)	0.547 (0.20–1.49)	0.23		
Diabetes	28 (26%)	21 (25%)	7 (32%)	1.40 (0.50–3.89)	0.52		
Obese (BMI ≥ 30)^b^	50 (54%)	38 (50%)	12 (71%)	2.40 (0.77–7.48)	0.12		
Weight ≥110 kg	19 (20%)	12 (15%)	7 (37%)	3.21 (1.05–9.80)	0.04		
Chronic non-infectious skin disorder	1 (1%)	1 (1%)	0	0.99 (0.97–1.01)	1.00		
Immunosuppressed at time of visit	2 (2%)	2 (2%)	0	0.98 (0.94–1.01)	1.00		
Provides healthcare to others	2 (2%)	1 (1%)	0	0.99 (0.97–1.01)	1.00		
MRSA phenotype	68 (65%)	55 (66%)	13 (59%)	0.87 (0.49–1.56)	0.65	0.42 (0.12–1.42)	0.16
MDR	29 (27%)	10 (12%)	19 (86%)	2.85 (1.07–7.62)	0.03		
Prior SSTI	35 (33%)	27 (32%)	8 (36%)	1.21 (0.45–3.20)	0.71		
Prior antibiotic history	16 (15%)	11 (13%)	5 (23%)	1.95 (0.60–6.36)	0.32		
Duration of infection prior to visit ≥7 days	48 (48%)	32 (40%)	16 (76%)	4.80 (1.59–14.41)	<0.01	6.02 (1.74–20.87)	<0.01
Severity							
Largest diameter ≥5 cm	49 (48%)	34 (42%)	15 (71%)	3.53 (1.24–10.02)	0.01	5.25 (1.58–17.42)	<0.01
Lesion area ≥25 cm^2^	37 (35%)	24 (29%)	13 (59%)	3.55 (1.34–9.39)	0.01		
Infection characteristics							
Erythema	78 (74%)	61 (74%)	17 (77%)	1.23 (0.40–3.72)	0.72		
Drainage	56 (53%)	45 (54%)	11 (50%)	0.84 (0.33–2.16)	0.72		
Ulceration	30 (29%)	22 (27%)	8 (36%)	1.58 (0.59–4.29)	0.43		
Abscess	76 (72%)	56 (67%)	20 (91%)	4.82 (1.05–22.14)	0.03		
Location							
Lower extremity	35 (33%)	26 (31%)	9 (41%)	1.54 (0.59–4.06)	0.38		
Head/neck/face	11 (10%)	10 (12%)	1 (5%)	0.35 (0.40–2.91)	0.45*		
Trunk	24 (23%)	17 (20%)	7 (32%)	1.84 (0.65–5.22)	0.26*		
Axilla	13 (12%)	11 (13%)	2 (9%)	0.66 (0.14–3.24)	0.61*		
Upper extremity	6 (6%)	5 (6%)	1 (5%)	0.75 (0.08–6.79)	1.00*		
Groin/buttock	17 (16%)	14 (17%)	3 (14%)	0.79 (0.21–3.03)	1.00*		
Treatment							
I&D only	4 (4%)	3 (4%)	1 (5%)	1.01 (0.91–1.12)	1.00		
I&D + antibiotics	57 (59%)	43 (51%)	14 (64%)	1.30 (0.68–2.51)	0.41		
Antibiotics only	32 (33%)	27 (36%)	5 (24%)	0.84 (0.63–1.13)	0.29		
Antibiotics							
Trimethoprim–sulfamethoxazole	81 (76%)	65 (77%)	16 (73%)	0.78 (0.27–2.27)	0.65		
Doxycycline	12 (11%)	9 (11%)	3 (14%)	1.32 (0.32–5.34)	0.71		
Clindamycin	7 (7%)	5 (6%)	2 (9%)	1.58 (0.26–8.75)	0.63		
Cephalexin	9 (9%)	7 (8%)	2 (9%)	1.10 (0.21–5.71)	1.00		
Discordant therapy	5 (5%)	3 (4%)	2 (11%)	2.82 (0.44–18.24)	0.26		

*Note* there were no cases of patients with the peripheral vascular disease, human immunodeficiency virus, cancer, and receipt of chemotherapy

*MRSA* methicillin resistant *S. aureus*, *MSSA* methicillin susceptible *S. aureus*, *SD* standard deviation, *OR* odds ratio, *CI* confidence interval, *aOR* adjusted odds ratio, *SSTI* skin and soft tissue infection, *BMI* body mass index, *I&D* incision and drainage

* Fishers exact test was used

Infection location, specific treatment strategies, or prescribed antimicrobial agents were not significantly associated with treatment failure. The proportion of discordant antimicrobial agents prescribed was higher in the treatment failure group than in the no-failure group, but did not reach statistical significance (11 vs. 4%, *p* = 0.20) which may be an artifact of the small sample size. MRSA isolates had significantly lower susceptibility to ciprofloxacin (*p* < 0.01) and erythromycin (*p* < 0.01). Two isolates (1 MRSA and 1 MSSA) were D-test positive.

**Fig. 1:**
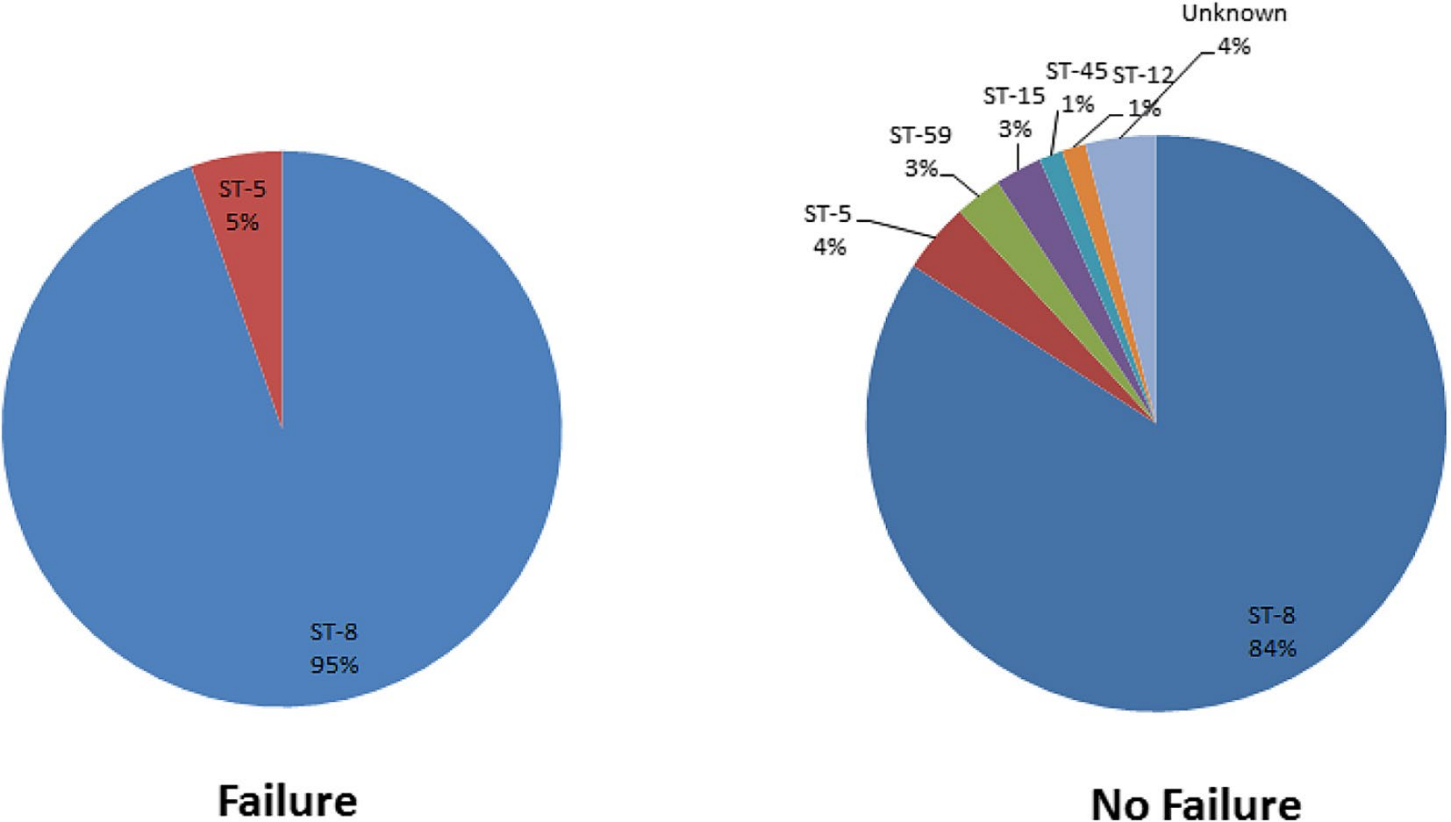
Multilocus sequence typing of *Staphylococcus aureus* isolates associated with treatment failure or no treatment failure

Molecular analysis (Fig. 1) was conducted on 98 isolates: 56% (65/116) of CA-MRSA and 43% (32/75) of CA-MSSA. All MRSA isolates and 68% of MSSA isolates were MLST strain type (ST) 8. Other MSSA strain types included: ST5, ST12, ST-15, ST-20, ST-45, and ST-59. Four isolates had undefined MLST designation. Furthermore, 95% of *S. aureus* SSTI treatment failures were ST-8 compared to 84% of cases with no treatment failure (*p* = 0.32).

## Discussion

Over the past 10 years, ambulatory care visits for SSTIs have increased exponentially. The worldwide emergence of CA-MRSA strains has made the management of *S. aureus* SSTIs extremely complicated and challenging [2, 3]. A clinical approach to the management of *S. aureus* SSTIs is to identify risk factors to predict those who are more likely to experience treatment failure.

Although it has been postulated that CA-MRSA related SSTIs may be associated with worse outcomes, SSTIs caused by MRSA did not have worse outcomes than those caused by MSSA in the South Texas community. Miller et al. found similar 30-day response rates among patients with CA-MRSA and CA-MSSA infection (23 [33%] of 70 vs. 13 [28%] of 47 patients; *p* = 0.55). In addition, patients with CA-MSSA infections were more likely to be re-hospitalized and to subjectively believe that they had not been cured [18]. Moreover, a previous randomized clinical trial of children with suspected *S. aureus* SSTIs found that the incidence of recurrence did not differ between children with MRSA vs. MSSA baseline infections [14]. Our findings further support the notion that the methicillin resistance phenotype is not a reliable predictor for clinical outcomes of community associated *S. aureus* infections. Rather, community associated *S. aureus* should be considered a single entity when evaluating virulence risks.

Identifying predictors for clinical failure can help target more aggressive treatment, monitoring, and decolonization procedures. This study identified that duration of infection 7 days and longer prior to the initial visit was the strongest predictor of treatment failure. This may be related to the natural course of infection that patients were presenting at a time of maximum intensity of inflammation and infection. Moreover, it may be that longer duration of an active *S. aureus* infection without proper treatment may have increased the likelihood for household or fomite transmission, both which have been shown to be important factors for infection recurrence [19–21]. This finding is in contrast to a recent study that found longer duration of symptoms was among the factors related to early response (at day 3). However, the investigation evaluated antibiotic response at day 3 among hospitalized SSTI patients; therefore, may not necessarily be associated with long-term outcomes including post-treatment failure and recurrence that were evaluated in the current study [7]. Importantly, this finding further supports the proposition that time to effective treatment is essential in the management of SSTIs, and possibly, that patients presenting with longer duration (≥7 days) of infections may require more aggressive measures and/or follow-up monitoring.

In addition, a lesion diameter of greater than 5 cm was associated with treatment failure. Lesion size has been used as a proxy for severity in several syndromes of skin infections, including necrotizing fasciitis and surgical site infections [22]. The 5 cm threshold used in this study was based on data from an observational study, in which abscesses larger than 5 cm were associated with treatment failure [23]. This investigation found a significant predictor of hospitalization on the first follow-up was having an infected area >5 cm in diameter at the initial evaluation (33% of patients with diameter >5 cm were later hospitalized vs. none with diameter ≤5 cm; *p* = 0.004). Other studies have also identified lesion size as an important indicator for severity and suggest treatment stratification approaches [24–26]. In the most recent guidelines for acute bacterial skin and skin structure infection (ABSSSI) clinical trials, the FDA recommends an evaluation of lesion size as a more quantitative assessment of infection severity and the change in lesion size at 48 to 72 h as the new primary endpoint [27]. Our findings provide further support to assess initial lesion sizes to predict infection severity and treatment outcomes.

Specific treatment strategies or type of prescribed antimicrobial agents were not found to be independent factors associated with treatment failure. It should be noted that because of the limited sample size, this study was not designed to perform direct comparisons of treatment approaches and antibiotic therapies. Larger investigations are needed to compare the role of treatment strategies and antibiotic regimens.

In our molecular analyses, ST-8 was the predominant strain type and was more likely to be found in patients whose treatment failed. However, this 11% absolute difference did not reach statistical significance due to the size of our cohort. This might suggest that the pathogenicity of community associated *S. aureus* may be based on the genetic background or lineage rather than the methicillin susceptibility phenotype. A more thorough investigation on the genomic characteristics of these strains is currently underway.

There are limitations to this study. First, we did not account for social and behavioral risk factors that may be associated with *S. aureus* SSTIs and/or clinical outcomes. Second, we used laboratory diagnosis to identify *S. aureus* cases. Patients presenting with SSTIs with no culture or were culture-negative may have different characteristics. The small sample size limited the ability to identify risks associated with lower exposures. Compliance of antimicrobials prescribed was not assessed. Finally, there may be limited generalizability to other regions outside of South Texas. To our knowledge, this is the first prospective study evaluating the clinical and epidemiological factors of *S. aureus* SSTI treatment failure in the primary care setting, adding important findings to the sparse literature in this growing population.

## Conclusions

The rates of treatment failure were similar among patients with CA-MRSA SSTIs to those with CA-MSSA SSTIs. Independent predictors for treatment failure included a duration of infection for greater than 7 days prior to the initial visit and a wound diameter of ≥5 cm. A heightened awareness of these risk factors could help direct clinical management and public health interventions in high-risk populations. Future large prospective investigations are required to validate these findings and to assess optimal treatment approaches.

## Abbreviations

SSTIs: skin and soft tissue infections
CA-MRSA: community-associated methicillin resistant *S. aureus*
CA-MSSA: community-associated methicillin susceptible *S. aureus*
STARNet: South Texas Ambulatory Research Network
PBRN: practice-based research network
MICs: minimum inhibitory concentrations
MDR: multidrug resistance
MLST: multilocus sequence type
CART: classification and regression tree
ORs: odds ratios
CIs: confidence intervals
aOR: adjusted odd ratio
SD: standard deviation
BMI: body mass index
I&D: incision and drainage
ST: strain type
